# Nodding syndrome (NS) and *Onchocerca Volvulus* (*OV*) in Northern Uganda

**DOI:** 10.11604/pamj.2017.28.1.13554

**Published:** 2017-09-04

**Authors:** David Kitara Lagoro, Denis Anywar Arony

**Affiliations:** 1 Gulu University, Faculty of Medicine, Department of Surgery, Gulu, Uganda; 2 Gulu University, Faculty of Medicine, Department of Biochemistry, Gulu, Uganda

**Keywords:** Nodding Syndrome, Onchocerca Volvulus, Uganda, Gulu

## Abstract

Nodding Syndrome (NS) is a childhood neurological disorder characterized by atonic seizures, cognitive decline, school dropout, muscle weakness, thermal dysfunction, wasting and stunted growth. There are recent published information suggesting associations between Nodding Syndrome (NS) with cerebrospinal fluid (CSF) VGKC antibodies and serum leiomidin-1 antibody cross reacting with *Onchocerca Volvulus* (*OV*). These findings suggest a neuro-inflammatory cause of NS and they are important findings in the search for the cause of Nodding Syndrome. These observations perhaps provide further, the unique explanation for the association between Nodding Syndrome and *Onchocerca Volvulus*. Many clinical and epidemiological studies had shown a significant correlation between NS and infestation with a nematode, *Onchocerca volvulus* which causes a disease, *Onchocerciasis*, some of which when left untreated can develop visual defect ("River Blindness"). While these studies conducted in Northern Uganda and Southern Sudan indicate a statistically significant association with (*OV* infection (using positive skin snips), we observe that (*OV* is generally endemic in many parts of Sub Saharan Africa and Latin America and that to date, no NS cases have been recorded in those regions. This letter to the Editor is to provide additional information on the current view about the relationship between Nodding Syndrome and *Onchocerca Volvulus* as seen in Northern Uganda.

## To the editors of the Pan African Medical Journal

There are recent published information suggesting associations between Nodding Syndrome (NS) with cerebrospinal fluid (CSF) VGKC antibodies [1] and serum leiomidin-1 antibody cross reacting with *Onchocerca Volvulus* ((*OV*) [2]. These findings suggest a neuro-inflammatory cause of NS and they are important findings in search for the cause of Nodding Syndrome [2]. These observations perhaps provide further, the unique explanation for the association between NS and (*OV* [1, 2]. Many clinical and epidemiological studies have shown a significant association between NS and infestation with a nematode, *Onchocerca volvulus* which causes a disease, *Onchocerciasis*, some of which when left untreated can develop visual defect ("River Blindness") [3, 4]. While many studies conducted in Northern Uganda and Southern Sudan indicate a statistically significant association with (*OV* infection (using positive skin snips), we observe that (*OV* is generally endemic in many parts of Sub Saharan Africa and Latin America and that to date, no Nodding Syndrome cases have been recorded in those regions [3-8]. The only WHO recognized locations in the world where NS has been confirmed are in Northern Uganda, South Sudan and Tanzania [3, 5-10]. The lack of NS outside these areas perhaps proposes a conclusion that (*OV* may perhaps not be a single cause of NS in Northern Uganda [3, 4-6, 8-10]. In addition, several case control studies conducted in (*OV* endemic areas shows that more than 10% of NS cases did not have OV(*OV* and conversely over 10% of the controls who had (*OV* did not have NS and so far there is no evidence that they developed NS [3-7]. Furthermore, the results of independent Polymerase Chain Reaction (PCR) analyses for (*OV* in the CSF of Sudanese and Tanzanian NS children was negative [3, 4, 8]. More still, it is reported that neurological illness (vertigo, headache and vomiting) in *Onchocerciasis* arises from the side effects of the anti-filarial drug treatment targeted at the microfilariae in cerebrospinal fluid which does not offer protection to head nodding [7, 9]. In addition, the peaks for the observed month of onset of nodding (April) does not correlate with the seasonal human biting activity of (*OV* infected black flies in the near Northeastern Uganda [8, 10]. This may suggest that (*OV* may perhaps not be the only contributory factor in NS but it may be partly contributing the stressor factor to such children with a possibly acquired disorder or a metabolic disorder (Kitara DL, personal communication).

**Figure 1:**
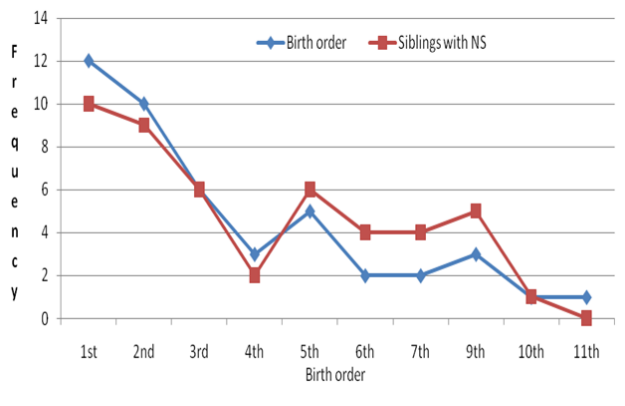
Shows pattern of NS occurrence and birth orders and other NS siblings in the family: the line graph (blue line) shows the birth order of NS children which was highest at 1^st^ birth and sloped down to the minimum at 4^th^ birth order and then two semi binomial peaks were observed at 5^th^ and 9^th^ birth orders. Therefore, birth order for these NS children indicates that they were more commonly found in the 1^st^, 2^nd^ and 3^rd^ birth order of the family (X^2^) = 9.68; p = 0.377. The second graph (Red lines) shows a near mirror image-like pattern of occurrence of NS among the other siblings in the family, following closely with the birth orders. There were more siblings who had NS in a family where there is the 1^st^ born having Nodding Syndrome and this association was observed to be statistically significant (X^2^) = 9.68; p = 0.004. In addition, one head of the household (we studied) had married 5 wives in the area and all the five wives had a NS child. This perhaps points towards a disease which is probably acquired and based in particular households. We propose that this was an acquired disease because none of the parents of NS children have clinical evidence of NS and neither do the offsprings of NS patients have been diagnosed with NS. Furthermore, no new cases of NS have been reported by the Ugandan Ministry of Health and World Health Organization since 2012 when all the IDP camps were disbanded and the communities resettled in their homes

**Figure 2:**
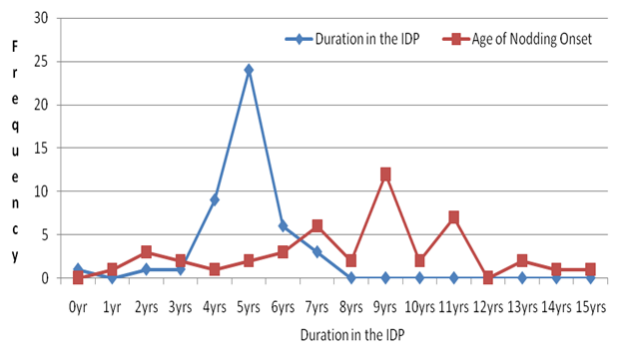
Shows the length of stay in IDPs in relation to the age of nodding onset in NS Children: the (blue line graph) for the duration of IDP camp stay shows a peak at 5 years (24/45) and slopes to zero by the 8^th^-9^th^year when the IDP camp had been disbanded while (Red line graph) shows the age of nodding onset which has three semi-peaks at 7 years (6/45), 9 years (12/45) and 11 years(7/45) respectively. All NS children were in IDP camps and that the majority (25/45) had spent 5 years in the IDP camps before onset of nodding. This perhaps indicates that the exposure factor of NS was in the IDPs camps (X^2^) = 22.146; p = 0.005

Further to this, (*OV* could be part of the aetiology of NS especially in the circumstance where the agent that transmits it to humans does transmit it with another agent (not yet described) simultaneously. In addition, there is another intriguing point about Nodding Syndrome in Northern Uganda that is, it clustered in space (occurring mainly discretely on either side of the Aswa and Pager rivers and their tributaries); time (IDP camp life) and person (onset mainly between 5-15 years) [4, 6]. This may perhaps point to the occurrence of the syndrome in relation to environmental and/or dietary factors [6]. The question which (*OV's* presence in NS children may not fully explain is the clustering of NS cases yet (*OV* occurs at endemic proportions in the areas where there are reported cases of NS [6]. It is the authors' view that (*OV* may perhaps be a biological agent with some links to NS and particularly when other mechanisms such as observed above can explain its roles in the pathophysiology of Nodding Syndrome [3-6]. In addition, findings on NS children observed that the majority were in the 1^st^, 2^nd^and 3^rd^ birth order and that most families that had a 1^st^ born child with NS, had more siblings in that family with NS than others (X^2^) = 9.68; p = 0.004 (Figure 1). These scenarios can perhaps point towards an acquired disease, possibly environmental or dietary factors that need further exploration. It is important to note at this point that Nodding Syndrome appears to be self-limiting, has no new cases of NS in this (*OV* endemic area of Northern Uganda since 2012 when the IDPs were disbanded and communities settled in their own homes and feeding on their home grown foods. In addition, Nodding Syndrome has not been seen in the parents and offsprings of NS patients in this region. Therefore, Nodding Syndrome may be a disorder which was likely to have been acquired in the IDP camps (Figure 2) or a disease which occurs as a result of a common environmental, dietary/nutritional exposure in IDP camps [3, 5]. The question that will remain unanswered in our minds though would be, why would an antibody to *Onchocerca Volvulus* which occurs in this area at endemic proportions have a clustered distribution?

## Conclusion

Nodding Syndrome is a childhood neurological disorder which affects communities in Northern Uganda and it is clustered in time (IDP camp stay), space (geographically located on either side of Pager and Aswa rivers) and person (onset mainly at 5-15 years). Several studies have shown an association between (*OV* and Nodding Syndrome however more investigations are required to confirm this correlation since NS occurs in (*OV* endemic area and that the occurrence of NS is clustered. In addition, there are no new cases of NS seen since 2012 when the IDPs were disbanded and communities resettled to their homes and feed on home grown foods.

## Competing interests

The authors declare no competing interest.
